# Homozygosity Mapping and Whole Exome Sequencing Reveal a Novel Homozygous *COL18A1* Mutation Causing Knobloch Syndrome

**DOI:** 10.1371/journal.pone.0112747

**Published:** 2014-11-13

**Authors:** Alireza Haghighi, Amit Tiwari, Niloofar Piri, Gudrun Nürnberg, Nasrollah Saleh-Gohari, Amirreza Haghighi, John Neidhardt, Peter Nürnberg, Wolfgang Berger

**Affiliations:** 1 Department of Genetics, Harvard Medical School, Boston, MA, United States of America; 2 Department of Medicine and the Howard Hughes Medical Institute, Brigham and Women’s Hospital, Boston, MA, United States of America; 3 Institute of Medical Molecular Genetics, University of Zurich Wagistrasse 12, CH-8952 Schlieren, Switzerland; 4 Kentucky Lions Eye Center, Department of Ophthalmology and Visual Sciences, University of Louisville, Louisville, KY, United States of America; 5 Cologne Center for Genomics (CCG), University of Cologne, Weyertal 115b, 50931 Cologne, Germany; 6 Genetic Department, Kerman University of Medical Sciences, Kerman, Iran; 7 Toronto General Hospital, University of Toronto, Toronto, Canada; 8 Zurich Center for Integrative Human Physiology (ZIHP), University of Zurich, Zurich, Switzerland; 9 Neuroscience Center Zurich (ZNZ), University and ETH Zurich, Zurich, Switzerland; 10 Center for Molecular Medicine Cologne (CMMC), University of Cologne, Robert-Koch Str. 21, D-50931 Cologne, Germany; 11 Cologne Excellence Cluster on Cellular Stress Responses in Aging-Associated Diseases (CECAD), University of Cologne, Joseph-Stelzmann-Str. 26, D-50931 Cologne, Germany; Eye Hospital, Charité, Germany

## Abstract

The aim of this study was to identify the genetic basis of a chorioretinal dystrophy with high myopia of unknown origin in a child of a consanguineous marriage. The proband and ten family members of Iranian ancestry participated in this study. Linkage analysis was carried out with DNA samples of the proband and her parents by using the Human SNP Array 6.0. Whole exome sequencing (WES) was performed with the patients’ DNA. Specific sequence alterations within the homozygous regions identified by whole exome sequencing were verified by Sanger sequencing. Upon genetic analysis, a novel homozygous frameshift mutation was found in exon 42 of the *COL18A1* gene in the patient. Both parents were heterozygous for this sequence variation. Mutations in *COL18A1* are known to cause Knobloch syndrome (KS). Retrospective analysis of clinical records of the patient revealed surgical removal of a meningocele present at birth. The clinical features shown by our patient were typical of KS with the exception of chorioretinal degeneration which is a rare manifestation. This is the first case of KS reported in a family of Iranian ancestry. We identified a novel disease-causing (deletion) mutation in the *COL18A1* gene leading to a frameshift and premature stop codon in the last exon. The mutation was not present in SNP databases and was also not found in 192 control individuals. Its localization within the endostatin domain implicates a functional relevance of endostatin in KS. A combined approach of linkage analysis and WES led to a rapid identification of the disease-causing mutation even though the clinical description was not completely clear at the beginning.

## Introduction

Knobloch syndrome (MIM:267750) (KS) is a very rare autosomal recessive developmental disorder. It is characterized by vitreoretinal degeneration usually with recurrent retinal detachment, retinitis pigmentosa-like features, lens subluxation, congenital high myopia, macular abnormalities and occipital encephalocele. The ocular features of the disease are similar to Stickler syndrome with optically empty vitreous and severe chorioretinal degeneration and high myopia, which is caused by mutations in collagen genes [1], [2].

Since its first introduction in 1971 by Knobloch and Layer, more than 50 cases have been reported all over the world. A defect in early cephalic neuroectodermal morphogenesis has also been suggested [1]. From 1971 to 1994, three families were reported from Hungary and the US, as well as a large Brazilian family with a history of consanguinity [1], [3], [4], all supporting the autosomal recessive inheritance of this condition. In 1996, homozygosity mapping in the same Brazilian family with 11 affected individuals assigned the KS gene to 21q22.3, close to the marker D21S17 [2]. Through a positional cloning approach, Sertie et al. showed that mutations in *COL18A1* are responsible for KS [5]. The gene is transcribed by use of two promoters and alternative splicing of the third exon [6]. The C-terminal part (183 amino acids), designated endostatin, can be cleaved-off proteolytically [7]. Collagen XVIII, encoded by the *COL18A1* gene, has an important role in determining the retinal structure as well as closure of the neural tube [5]. This protein is an essential component of the basement membrane of the iris, vitreous and retina and its presence is critical for normal eye development during embryogenesis. Collagen XVIII also seems to play important functional roles in neuronal cell migration and as a component of the basement membrane of kidney as well as teeth development [8], [9]. It has been shown in an experimental model that collagen XVIII is also important for maintaining capillary permeability in striated muscle [10]. Williams et al. reported a case of KS with bilateral renal anomalies, which implies a role of collagen XVIII in kidney physiology [11]. In 1998, Wilson et al. reported two siblings from New Zealand and proposed a possible involvement of mesoderm in morphogenesis as those patients had abnormal pulmonary lymphatics [12]. Sniderman et al. reported a case with anterior midline scalp defect reflecting further clinical variability in this rare autosomal recessive syndrome [13].

Mutations in *COL18A1* gene can lead to occipital encephalocele and severe ocular alterations [14]. It was also shown that a lack of either the short (NC1-303) or long isoform (NC1-728) of collagen XVIII causes similar phenotypes, but patients lacking all isoforms exhibit increasingly severe ocular alterations. This suggests that both isoforms play critical roles in the maintenance and organization of the human eye [14]. In 2003, Kliemann et al. reported neuronal migration disorders in two Brazilian patients for the first time [15]. This was followed by the report of a case with persistent fetal vasculature and initiated the discussion of a possible role of endostatin in vascular remodeling of the fetal eye [16]. In two siblings with KS from France, one presented with mental retardation and severe supratentorial CNS anomalies, and a second fetus with severe brain malformations, complete vermian agenesis, and mesencephalic hamartoma, suggested that either endostatin or full-length collagen XVIII play a role in neuronal migration, revealing that CNS anomalies in KS were more severe than initially thought [17]. In addition, Knobloch syndrome was found to be associated with acute lymphoblastic leukemia in an El Salvadorian patient. The authors proposed that an increased risk of cancer may be an endostatin associated effect and suggested monitoring of KS cases with respect to leukemia or other cancers [18].

Here, we report an Iranian family in which the proband was initially diagnosed with congenital chorioretinal degeneration and myopia. A combination of homozygosity mapping and whole exome sequencing (WES) identified a novel mutation in *COL18A1* and led to the diagnosis of Knobloch syndrome. This is the first case of KS identified using such an approach. It is also the first KS case found in a family of Iranian descent.

## Materials and Methods

### Enrollment of participants and clinical examinations

Recruitment of the family was based on interviews, questionnaires, and clinical examination of affected and unaffected individuals by ophthalmologists and geneticists. An informed written consent for clinical and molecular investigation was obtained from all family members. The study was conducted in accordance with the Helsinki Declaration. The approval for genetic testing was awarded to The Institute of Medical Molecular Genetics by the Federal Office of Public Health (FOPH) in Switzerland. Complete ophthalmic examination was performed. Visual acuity was measured using the Snellen chart. Ophthalmological exam was performed using the slit lamp biomicroscope, indirect biomicroscopy and indirect ophthalmoscopy. Intraocular pressure was measured by the Goldman tonometer. Flash ERG was performed to evaluate the overall photoreceptor function.

### DNA extraction

Peripheral blood samples were collected from all family members. DNA was extracted using QIAamp DNA blood Midi kits (Qiagen, Hilden, Germany).

### Genotyping and linkage analysis

Whole genome genotyping for the proband and her parents was carried out using the Genome-Wide Human SNP Array 6.0 (Affymetrix, Santa Clara, CA). Data were analyzed using the program Graphical Representation of Relationships (GRR) [19]. Linkage analysis was performed assuming autosomal recessive inheritance, full penetrance, consanguinity and a mutation carrier frequency of 0.0001. Multipoint LOD scores were calculated using the program ALLEGRO [20]. All data handling was done using the graphical user interface ALOHOMORA [21]. Homozygous genomic regions restricted to the patient and absent in her parents were identified and a tab-delimited “regions of interest” file was generated.

### Whole exome sequencing analysis

Whole exome sequencing was performed using NimbleGen SeqCap EZ Human Exome Library (Roche NimbleGen Inc., Madison, WI) for library preparation and paired-end 100 nt sequencing on Illumina HiSeq Alignment of sequence reads, indexing of the reference genome, variant calling and annotation was done with a pipeline based on BWA [22], Samtools [23], Picard and Annovar [24]. Variants were annotated using Alamut-HT (Interactive Biosoftware, Rouen, France) and filtered against the above described regions of interest file in order to obtain variants within homozygous regions specific to the patient. Variants were visualized on Alamut Viewer 2.2 (Interactive Biosoftware, Rouen, France). A filtering pipeline was established to remove known SNPs or benign polymorphisms. The following variants passed the filter: (a) non-annotated novel SNPs (b) variants with minor allele frequency ≤0.02 (c) variants with a SIFT score of ≤0.05 (deleterious) as well as (d) variants with a MAPP (Multivariate Analysis of Protein Polymorphism) -score ( = bad or unknown).

### Primer design, PCR amplification and Sanger sequencing

Primers were designed using Primer3 software [25] and purchased at Microsynth AG (Balgach, Switzerland). Exon 42 of *COL18A1* was amplified in duplicate from genomic DNA of the patient and available family members using Hot FirePol DNA Polymerase (Solis BioDyne, Tartu, Estonia) and the following primers: forward 5′-GTGTCTGGCAGAAGCAGCAT-3′ and reverse 5′-TCACAGGTCAGGGGAGAGTT-3′. Sanger sequencing was performed using the Big Dye Terminator Cycle v1.1 Sequencing Kit (Applied Biosystems, Carlsbad, California, USA) and ABI Prism 3730 Genetic Analyzer (Applied Biosystems, Carlsbad, California, USA). 192 randomly collected DNAs from the general population were used to assess the frequency of the mutation. Sanger sequencing data was analyzed using SeqScape v2.6 (Applied Biosystems, Carlsbad, California, USA).

## Results

### Clinical Description

**Figure 1:**
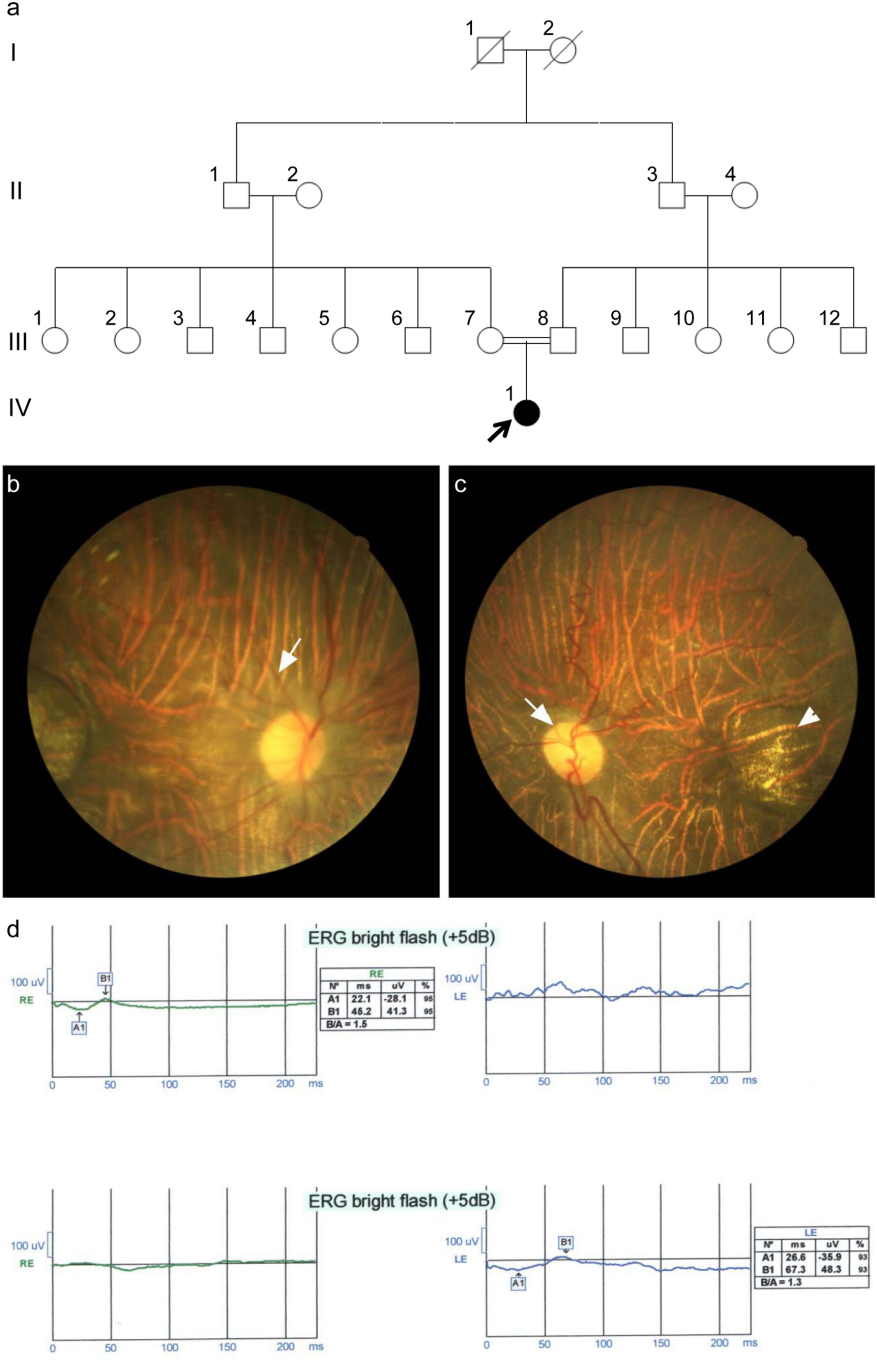
a. Pedigree of a consanguineous family initially diagnosed with chorioretinal degeneration and high myopia (in some family members). b. Color fundus photo OD shows the diffuse chorioretinal atrophy (white arrow points to the arterial narrowing). c. Color fundus photo OS showing the waxy optic disc (white arrow) and the well-circumscribed macular atrophic lesion (arrowhead). d. Flash ERG OU demonstrates severely diminished amplitudes of the a and b waves.

The female proband (IV-1, Figure 1a) was examined at 10 years of age. She initially presented at eight months of age with deviation and involuntary eye movements. On physical examination, at the first visit, she had nystagmus and esotropia, and normal anterior segment oculus uterque (both eyes, OU) [specifically speaking iris was normal OU and after pupillary dilation there was no abnormality in lens periphery OU]. Cycloplegic refraction revealed highly myopic refractive error OU (−16.00 D). Dilated fundus examination showed waxy optic discs (Figure 1c-white arrow) with a cup-to-disc ratio of 0.1, arterial narrowing (Figure 1b-white arrow) and a diffuse chorioretinal atrophy with a well-defined central atrophic lesion in the center of the macula OU (Figure 1c-arrowhead).

Her parents were first cousins and there was no history of any similar condition in the family, apart from the high myopia in two of the aunts (III-10: −8.00 D, OU with myopic chorioretinal atrophy and III-11: −16.00 D: OD; −9.50–2.00×85°:OS with high myopic chorioretinal atrophy) (Figure 1a).

Flash electroretinography (ERG), demonstrated severely diminished amplitude of a and b waves OU which was in favor of both cone and rod dysfunction (Figure 1d).

Based on clinical findings differential diagnosis included the following:

1. Congenital-onset central chorioretinal dystrophy associated with high myopia [26], this has been reported in a family in middle east without genotyping; however ocular features are very similar to our patient
2. Leber’s congenital amaurosis type with central macular atrophy (NMNAT1 mutation) [27]. Ocular findings of this type of leber’s is also very similar to what we saw in our patient

Eye glasses were prescribed for the patient and she was then followed up somewhere else for seven years. At 8 years of age, her physical and intellectual developments were normal. At school, she maintained good academic standing, with the assistance of low-vision aids such as magnifiers or magnified prints. On ophthalmic examination, nystagmus was apparent. The best corrected visual acuity (BCVA) was 20/400 OU and the refractive error was −12.50 D, OU. Dilated fundus examination was remarkable for waxy optic nerve with a cup-to-disc ratio of 0.1, arterial narrowing and diffuse chorioretinal atrophy (myopic changes) with a well-defined central atrophic lesion with prominent underlying large choroidal vessels. The clinical findings were the same as seven years ago.

A few months later, she presented with a chief complaint of visual field defect in the left eye for a few days. Fundus examination revealed a superonasal retinal detachment. She underwent 23-gauge pars plana vitrectomy (PPV) and silicone oil injection. After 2 months, she developed cataract (as a consequence of vitrectomy surgery); however, the retina was flat. Cataract surgery and posterior chamber intraocular lens implantation (PC IOL) were performed accompanied with removal of silicone oil through a separate scleral incision. BCVA after the surgery was 20/400. Two years later, she developed posterior capsule opacity, which required YAG laser capsulotomy to restore the vision.

Ophthalmic examination of the parents was unremarkable with normal vision and extraocular movements, and normal anterior and posterior segments. The proband’s two aunts (III-10 and III-11, Figure 1a) had high myopia OU. Anterior segments were normal OU. Fundus examination revealed chorioretinal atrophy (myopic changes) without unusual findings.

### Genetic Analysis

#### Genotyping and linkage analysis

Genome-wide genotyping of the patient-parent-trio with a high-density SNP array identified19 homozygous regions that were unique to the proband and not observed in her parents (table S1, figure S1). The LOD (logarithm of odds) scores obtained for these homozygous regions were not higher than 1.8 (figure S1) as expected for a trio with consanguineous marriage.

#### Whole Exome Sequencing

**Figure 2:**
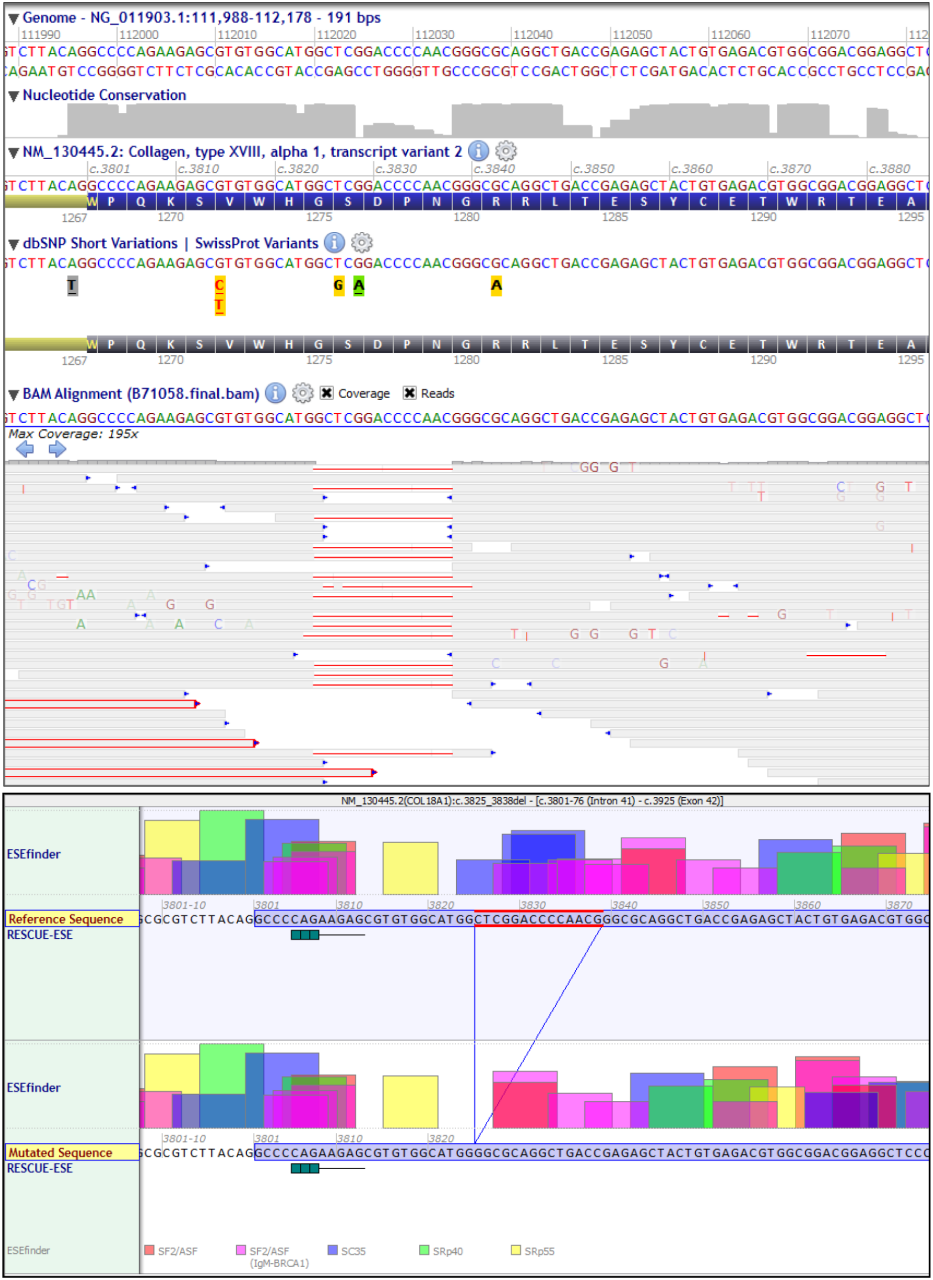
Coverage of sequencing reads in *COL18A1* including a 14 bp deletion (NM_130445.2:c.3825_3838del:p.Ser1276Alafs*9 (Exon 42) as seen on Alamut Viewer 2.2.0 (Upper panel). A loss of multiple strong ESEs is predicted due to the deletion (Lower panel).

In total, 8.87 Gb of data were obtained upon sequencing that constituted about 9.5*10^7^ reads. While mean coverage was 98x, 97% of sequences had at least 10-times coverage and 90% of the sequences were covered at least 30-times. 38276 variants were obtained from the whole-exome sequencing of the patient’s DNA and alignment to the reference sequence. Using the “regions of interest” file (see Materials and Methods), variants were annotated by using Alamut-HT. 2327 variants were obtained in the homozygosity regions of interest with 219 of them being novel (not annotated in dbSNP). Almost one-fifth of the variants were missense mutations, 114 of them predicted to be damaging. Table 1 lists the distribution of the sequence variations. We subjected the 2327 variants to our filtering pipeline (described in Materials and Methods) and found a novel deletion in *COL18A1*: NM_130445.2:c.3825_3838del:p.Ser1276Alafs*9. This 14 bp deletion is predicted to cause a frameshift at a highly conserved Serine residue at position 1276 (Figure 2) and results in a stop codon 9 triplets downstream. Snapshots of Alamut Viewer 2.2 in figure 2 show the bidirectional coverage of the sequencing reads where a deletion in exon 42 of *COL18A1* gene can be clearly seen (upper panel). The deletion is also predicted to lead to strong loss of multiple exonic splicing enhancers (Figure 2, lower panel).

**Table 1:** Classification of the type of homozygous variants obtained by linkage analysis and whole exome sequencing.

Type of sequence alteration	SIFT prediction	Number	Annotated SNPs	Novel variants
**Nonsense (stop) mutation**	-	2	2	0
**Amino acid substitution**	damaging	114	82	28
**Amino acid substitution**	tolerated	378	357	21
**Silent mutation**	-	685	629	56
**Intronic or UTR mutation**	-	1031	924	107
**Frameshift deletion**	-	4	3	1
**Frameshift insertion**	-	2	2	0
**In-frame deletion**	-	5	5	0
**In-frame duplication**	-	4	3	1
**In-frame insertion**	-	1	1	0
**Upstream and downstream mutations**	-	101	96	5

#### Segregation Analysis

**Figure 3:**
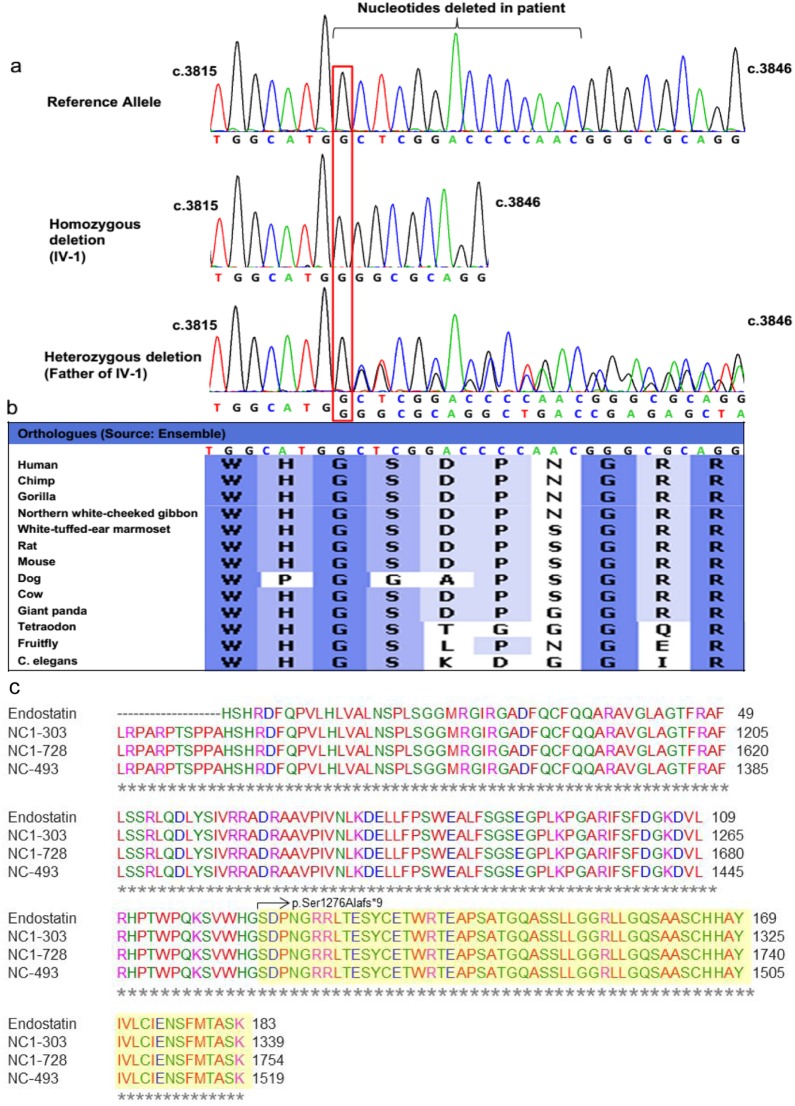
a. Sanger sequencing of *COL18A1* mutation NM_130445.2:c.3825_3838del:p.Ser1276Alafs*9 (Exon 42) comparing a wild type sequence (top), homozygous deletion in index patient IV-1 (middle) and heterozygous deletion in father of the index patient (bottom). b. Conservation of the deleted nucleotides across various species. c. Alignment of three isoforms of *COL18A1* and endostatin. The highlighted region is deleted in patient IV-1.

The family of the proband spanned four generations and comprises 19 family members. The parents of the index patient were first cousins. The proband and ten other family members were involved in this study (Figure 1a). Subjects were genotyped by direct Sanger sequencing of the mutation in *COL18A1* (NM_130445.2:c.3825_3838del:p.Ser1276Alafs*9). Sanger sequencing confirmed the exome sequencing results that the proband (IV-1, Figure 1a) was homozygous for this mutation (Figure 2 and 3a). Parents of the proband (III-7 and III-8, Figure 1a) were heterozygous for the same mutation (Figure 3a), so were two uncles (III-3 and III-4) and two aunts (III-5 and III-11) (data not shown). Three uncles (III-6, III-9 and III-12, Figure 1a) and one aunt (III-10, Figure 1a) did not carry this mutation (data not shown). This frameshift deletion cosegregated with the phenotype in the family as an autosomal recessive trait as expected for Knobloch syndrome. The mutation was absent in control DNA samples of Caucasian origin from 192 unrelated individuals tested for this mutation (data not shown). Controls from Iranian descent were not available. Two of the aunts (III-10 and III-11, Figure 1a) also showed high myopia but this condition did not cosegregate with the identified *COL18A1* frameshift deletion. III-10 was homozygous for the reference allele and III-11 was heterozygous for the mutation but both had high-grade myopia.

## Discussion

The proband is the first case of Knobloch syndrome in a family of Iranian descent. Twenty mutations leading to KS have been described in the *COL18A1* gene. This includes 5 missense mutations [12], [14], [15], [28]–[30], 3 mutations affecting correct splicing [5], [17], [31], 5 small deletions [14], [28], [31], [32], 4 small insertions [14], [28], [31], [33], 2 gross deletions [28], [29], [31] and 1 small indel [29] (Source: HGMD Professional 2014.2).

Since the patient was an offspring of a first cousin marriage, we employed a combined approach of homozygosity mapping and whole exome sequencing and identified the disease-causing mutation using only a fraction of the time and costs in comparison to that required by conventional analysis. By filtering the exome data with our filtering pipeline (described in Materials and Methods), only a single sequence variant in the *COL18A1* gene remained as a mutation candidate associated with the disease.

The patient was initially clinically characterized with congenital-onset central chorioretinal dystrophy and myopia. Since we found a mutation in *COL18A1,* a gene that had been previously associated with Knobloch syndrome, we reanalyzed the clinical data in depth. Retrospective evaluation of additional clinical data upon identification of the mutation in *COL18A1* revealed that the patient had a meningocele surgically removed at 6 weeks of age. Thus the clinical picture of the patient is consistent with Knobloch syndrome. Mutations in *COL18A1* are specific to Knobloch Syndrome and no additional gene has been identified so far [29]. The phenotype includes retinal detachment, nystagmus and congenital meningocele. While vitreoretinal degeneration is the more common phenotype seen in KS patients, our patient showed chorioretinal degeneration, a rather uncommon phenotype, which has been previously reported by Mahajan et al. 2010 [18]. Two of the family members of the patient had extreme myopia, which is a phenotype frequently observed in KS patients. However, this did not segregate with the *COL18A1* mutation in the family.

The spectrum of ophthalmologic findings in KS was documented in a recent report by Khan et al [34] in eight patients. Six of their patients had ectopia lentis, which was not seen in our case; however their retinal findings are very similar to our case. We suggest that in patients with congenital high myopia, retinitis pigmentosa like chorioretinal atrophy and a well-defined atrophic macular lesion, Knobloch syndrome should be considered as the most likely diagnosis. It seems that anterior segment findings are less consistent. These cases are at a high risk of retinal detachment at young age and should be carefully observed.

Collagen XVIII is encoded by three isoforms, each differing in their N-terminal regions. The mutation described in this study, c.3825_3838del, causes a frameshift deletion in the last exon of *COL18A1*, thus leading to an incomplete mRNA. This region of *COL18A1* also encodes for a cleavable protein called endostatin that has been shown to be anti-angiogenic and inhibit tumor formation [7]. The identified mutation causes loss of the terminal 60 amino acids of endostatin (Figure 3c, truncated amino acids are highlighted in yellow). This immediately points to a role of endostatin in the disease physiology of KS. Endostatin is encoded by all isoforms of *COL18A1*, therefore, a mutation in the domain containing endostatin will affect all isoforms of *COL18A1*. Previous studies have shown a severe loss of endostatin levels in KS patients [14], [31]. Due to poor health condition of the proband, we could not obtain additional samples for verifying her endostatin levels.

Fukai et al. showed that mice lacking collagen XVIII/endostatin manifest developmental eye defects, e.g. lack or abnormal outgrowth of retina, delayed regression of blood vessels in the vitreous along the surface of retina after birth and reduced expression of VEGF (Vascular Endothelial Growth Factor) [35]. Persistent fetal vasculature is the other endostatin-deficiency related condition in KS patients. Loss of endostatin or its deficiency might cause delayed or reduced fetal blood vessel regression in the eye. This can cause failure of normal vascular development in the retina [16]. Endostatin physically interacts with extracellular matrix components such as laminin-1, fibulin-1, fibulin-2, fibronectin, heparin sulfate, nidogen-2 and perlecan [16], [31], [36], [37]. Mutations in collagen XVIII/endostatin that lead to loss of the protein/protein function might thus cause changes in the overall structural organization and stability of the extracellular matrix. Therefore, phenotypic variations of mutations in collagen XVIII could be either due to reorganization of the ECM (structural changes) or through defects in angiogenesis during eye development.

This study describes a novel KS-causing mutation in *COL18A1*. Our results also suggest a role of endostatin in the physiopathology of KS. In the genetic analysis of this case, even though the LOD scores obtained upon linkage analysis were not significant (Significance ≥3, figure S1), using the relatively small homozygous regions, we were able to detect the disease-causing mutation upon whole exome analysis. Identification of the mutation using this combined approach can facilitate the confirmation of the clinical diagnosis when the initial clinical picture is not fully clear. In our case, the initial clinical diagnosis was congenital-onset central chorioretinal dystrophy and myopia. Identification of a mutation in *COL18A1* led to a retrospective clinical analysis upon which the clinical diagnosis was confirmed to be Knobloch syndrome. With different medical systems across the world and increased mobility of human beings to different countries and geographical areas, a complete clinical record is not always available to clinicians. Molecular analysis tools such as that described is this study can provide the necessary support for a precise clinical characterization and diagnosis of the disease. This may contribute to application of correct treatment and or medical care regimen. In addition, this approach led to identification of the underlying causative mutation using a fraction of the costs and time in comparison to the conventional candidate gene approaches. Thus, it presents a strong case for using a combined approach of linkage analysis (homozygosity mapping) with exome sequencing for rapid and cost-effective diagnosis of Mendelian diseases.

## Supporting Information

Figure S1 **Genome-wide linkage analysis: Parametric linkage analysis of the family was performed with 20,044 selected SNP markers from the Affymetrix SNP Array 6.0.** LOD scores (y-axis) were calculated using ALLEGRO and plotted against the genetic distance in cM (centi Morgan) on the x-axis, which is used as a surrogate for the genomic position. Chromosomes are concatenated from p-ter to q-ter from left to right.
(TIF)

Table S1 **Homozygous regions in patient IV-1 obtained by linkage analysis and subtracting common homozygous regions from parents.**
(DOCX)
