# Supplementary figures and images for: Homozygosity Mapping and Whole Exome Sequencing Reveal a Novel Homozygous COL18A1 Mutation Causing Knobloch Syndrome
Source: PLoS One. 2014 Nov 13;9(11):e112747. doi: 10.1371/journal.pone.0112747 (PMC4231049; doi:10.1371/journal.pone.0112747)

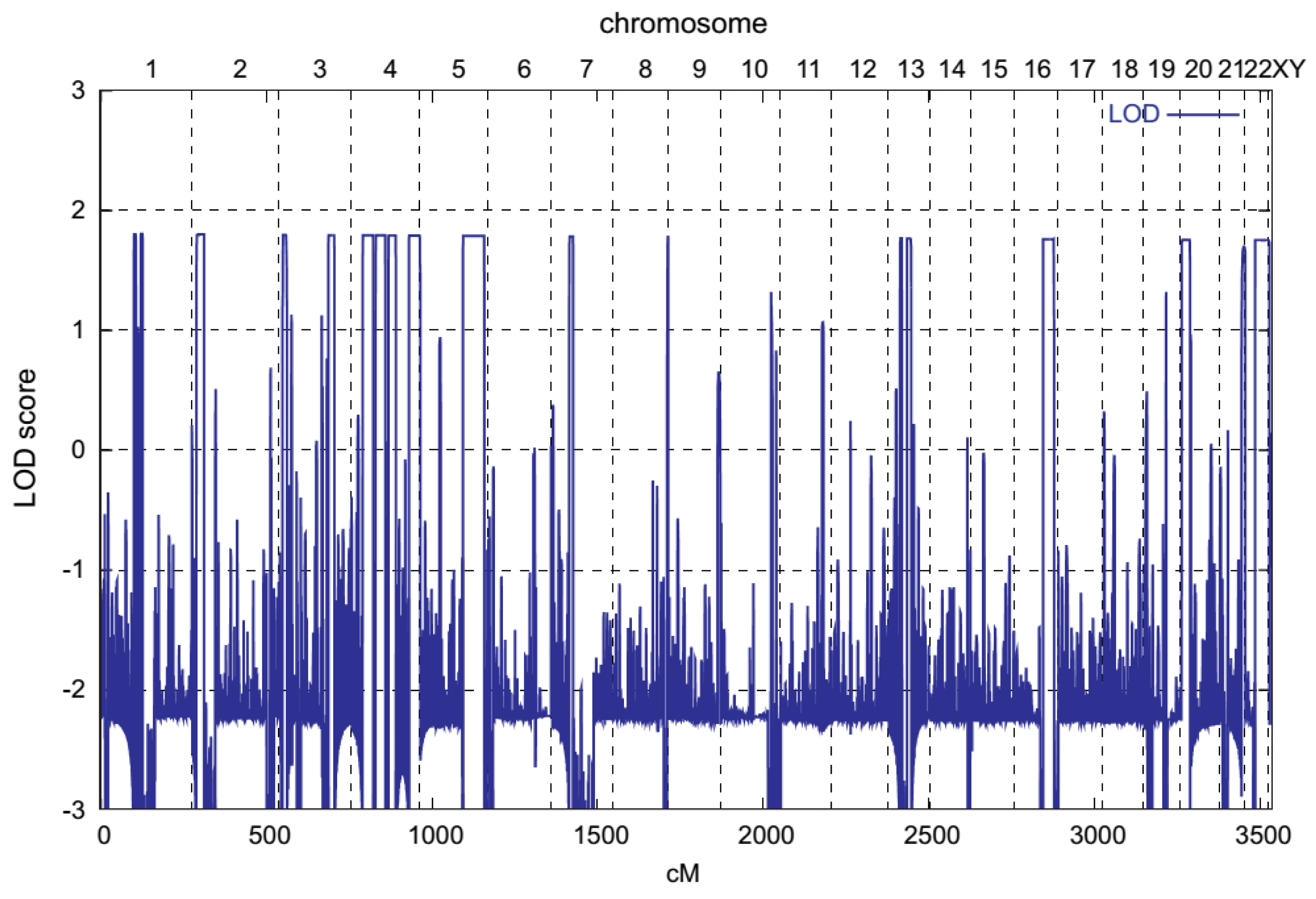

Supplement: Figure S1 — Genome-wide linkage analysis: Parametric linkage analysis of the family was performed with 20,044 selected SNP markers from the Affymetrix SNP Array 6.0. LOD scores (y-axis) were calculated using ALLEGRO and plotted against the genetic distance in cM (centi Morgan) on the x-axis, which is used as a surrogate for the genomic position. Chromosomes are concatenated from p-ter to q-ter from left to right. (TIF) [file pone.0112747.s001.tif]
