# Supplementary material for: Homozygosity Mapping and Whole Exome Sequencing Reveal a Novel Homozygous COL18A1 Mutation Causing Knobloch Syndrome
Source: PLoS One. 2014 Nov 13;9(11):e112747. doi: 10.1371/journal.pone.0112747 (PMC4231049; doi:10.1371/journal.pone.0112747)
Supplement: Table S1 — Homozygous regions in patient IV-1 obtained by linkage analysis and subtracting common homozygous regions from parents. (DOCX) [file pone.0112747.s002.docx]

| **Chromosome** | **Start position** | **End Position** |
| --- | --- | --- |
| chr1 | 75992462 | 82590298 |
| chr1 | 99963687 | 107376059 |
| chr2 | 6434587 | 16752999 |
| chr3 | 4237348 | 9232109 |
| chr3 | 143758399 | 168029028 |
| chr4 | 17195632 | 23265535 |
| chr4 | 23352070 | 44372045 |
| chr4 | 57937117 | 95551967 |
| chr4 | 108035955 | 137628416 |
| chr4 | 178295429 | 190915650 |
| chr5 | 125385126 | 174597723 |
| chr7 | 35582627 | 44827517 |
| chr8 | 143570921 | 146293414 |
| chr13 | 37293677 | 40940726 |
| chr13 | 60088876 | 74630351 |
| chr16 | 69534636 | 84894281 |
| chr20 | 69408 | 7929520 |
| chr21 | 45724055 | 48077812 |
| chr22 | 28130072 | 51175626 |

Supplementary Table 1. Homozygous regions in patient IV-1 obtained by linkage analysis and subtracting common homozygous regions from the parents
